# 3-(Adamantan-1-yl)-4-methyl-1-({4-[3-(tri­fluoro­meth­yl)phen­yl]piperazin-1-yl}meth­yl)-4,5-di­hydro-1*H*-1,2,4-triazole-5-thione

**DOI:** 10.1107/S1600536813009495

**Published:** 2013-04-13

**Authors:** Ali A. El-Emam, Abdul-Malek S. Al-Tamimi, Khalid A. Alrashood, Seik Weng Ng, Edward R. T. Tiekink

**Affiliations:** a Department of Pharmaceutical Chemistry, College of Pharmacy, King Saud University, Riyadh 11451, Saudi Arabia; b Department of Pharmaceutical Chemistry, College of Pharmacy, Salman bin Abdulaziz University, Alkharj 11942, Saudi Arabia; c Department of Chemistry, University of Malaya, 50603 Kuala Lumpur, Malaysia; d Chemistry Department, Faculty of Science, King Abdulaziz University, PO Box 80203 Jeddah, Saudi Arabia

## Abstract

In the title compound, C_25_H_32_F_3_N_5_S, two independent mol­ecules comprise the asymmetric unit and are related across a pseudo-centre of inversion. The piperazine rings have chair conformations with each N-bound substituent occupying an equatorial position so that the dihedral angles between the planes of the triazole and benzene ring are 78.20 (19) and 79.10 (19)° for the two independent mol­ecules, indicating that the mol­ecules have an L-shape. In the crystal, a three-dimensional architecture is stabilized by C—H⋯π inter­actions. The crystal studied was an inversion twin with the fractional contribution of the minor component being 0.27 (9).

## Related literature

For the diverse biological activities of adamantane derivatives, see: Al-Deeb *et al.* (2006 ▶); Al-Omar *et al.* (2010 ▶). For a related adamantanyl structure, see: El-Emam *et al.* (2012 ▶).
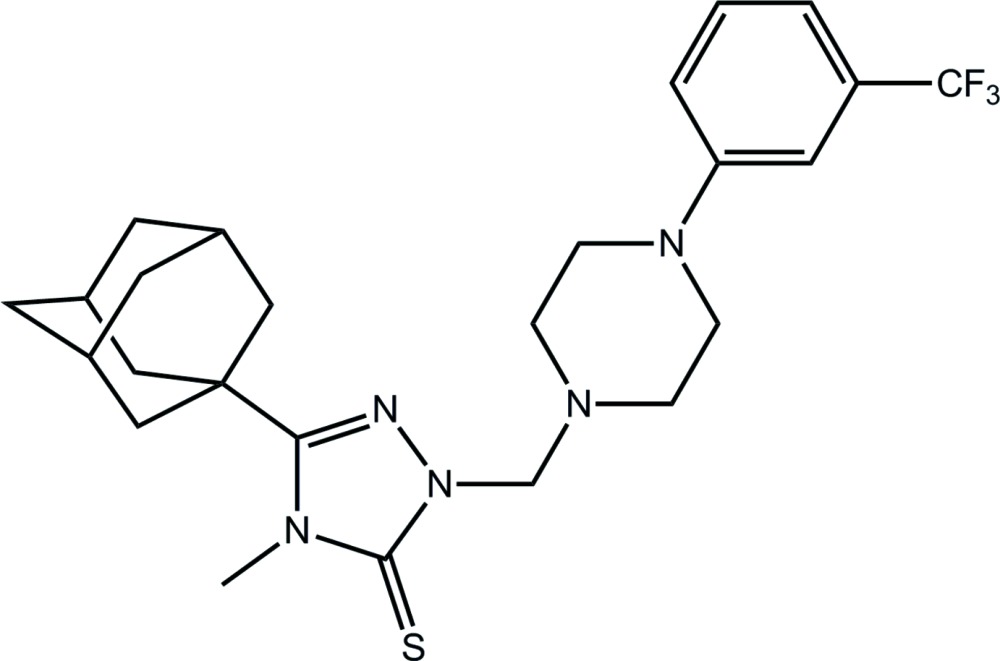


## Experimental

### 

#### Crystal data

C_25_H_32_F_3_N_5_S
*M* *_r_* = 491.62
Orthorhombic, 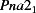

*a* = 28.8100 (15) Å
*b* = 6.6052 (4) Å
*c* = 25.7717 (14) Å
*V* = 4904.2 (5) Å^3^
*Z* = 8
Mo *K*α radiation
μ = 0.18 mm^−1^
*T* = 295 K
0.40 × 0.30 × 0.20 mm

#### Data collection

Agilent SuperNova Dual diffractometer with an Atlas detector
Absorption correction: multi-scan (*CrysAlis PRO*; Agilent, 2011 ▶) *T*_min_ = 0.878, *T*_max_ = 1.000
19420 measured reflections
9351 independent reflections
6701 reflections with *I* > 2σ(*I*)
*R*_int_ = 0.038

#### Refinement

*R*[*F* ^2^ > 2σ(*F* ^2^)] = 0.058
*wR*(*F* ^2^) = 0.151
*S* = 1.03
9351 reflections
616 parameters
1 restraint
H-atom parameters constrained
Δρ_max_ = 0.37 e Å^−3^
Δρ_min_ = −0.28 e Å^−3^
Absolute structure: Flack (1983 ▶), 3730 Friedel pairs
Flack parameter: 0.27 (9)

### 

Data collection: *CrysAlis PRO* (Agilent, 2011 ▶); cell refinement: *CrysAlis PRO*; data reduction: *CrysAlis PRO*; program(s) used to solve structure: *SHELXS97* (Sheldrick, 2008 ▶); program(s) used to refine structure: *SHELXL97* (Sheldrick, 2008 ▶); molecular graphics: *ORTEP-3 for Windows* (Farrugia, 2012 ▶), *QMol* (Gans & Shalloway, 2001 ▶) and *DIAMOND* (Brandenburg, 2006 ▶); software used to prepare material for publication: *publCIF* (Westrip, 2010 ▶).

## Supplementary Material

Crystal structure: contains datablock(s) global, I. DOI: 10.1107/S1600536813009495/hg5307sup1.cif

Structure factors: contains datablock(s) I. DOI: 10.1107/S1600536813009495/hg5307Isup2.hkl

Supplementary material file. DOI: 10.1107/S1600536813009495/hg5307Isup3.cml

Additional supplementary materials:  crystallographic information; 3D view; checkCIF report

## Figures and Tables

**Table 1:** Hydrogen-bond geometry (Å, °) *Cg*1–*Cg*4 are the centroids of the N1–N3,C2,C3, C19–C24, N6–N8,C27,C28 and C44–C49 rings, respectively.

*D*—H⋯*A*	*D*—H	H⋯*A*	*D*⋯*A*	*D*—H⋯*A*
C6—H6⋯*Cg*2^i^	0.98	2.95	3.817 (4)	148
C13—H13*B*⋯*Cg*3^ii^	0.97	2.86	3.782 (4)	158
C31—H31⋯*Cg*4^iii^	0.98	2.94	3.873 (5)	159
C38—H38*B*⋯*Cg*1^iv^	0.97	2.97	3.723 (5)	135
C45—H45⋯*Cg*2^v^	0.93	2.97	3.708 (5)	137

Symmetry codes: (i) 
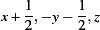
; (ii) 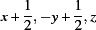; (iii) 
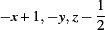
; (iv) 
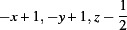
; (v) 
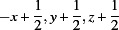
.

**Fig. 1.**
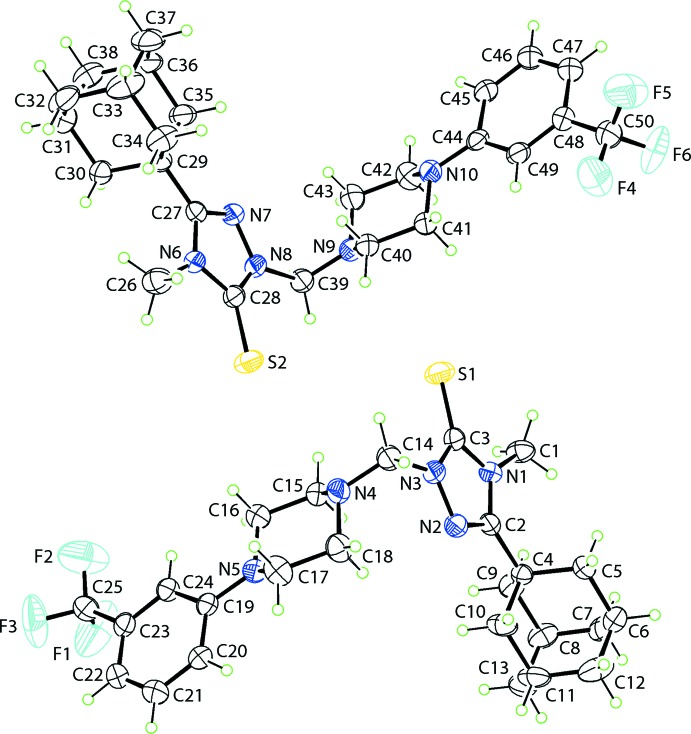
The molecular structures of the two independent molecules comprising the asymmetric unit of (I) showing the atom-labelling scheme and displacement ellipsoids at the 35% probability level.

**Fig. 2.**
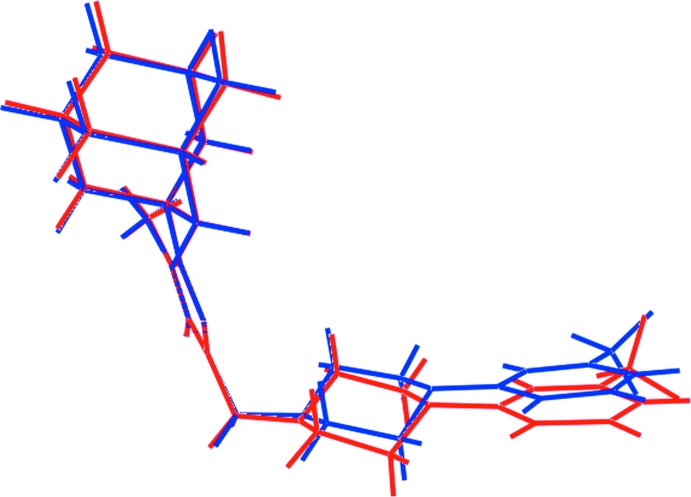
Overlay diagram of the S1- (red image) and inverted S2-containing molecules (blue) where the triazole rings have been superimposed.

**Fig. 3.**
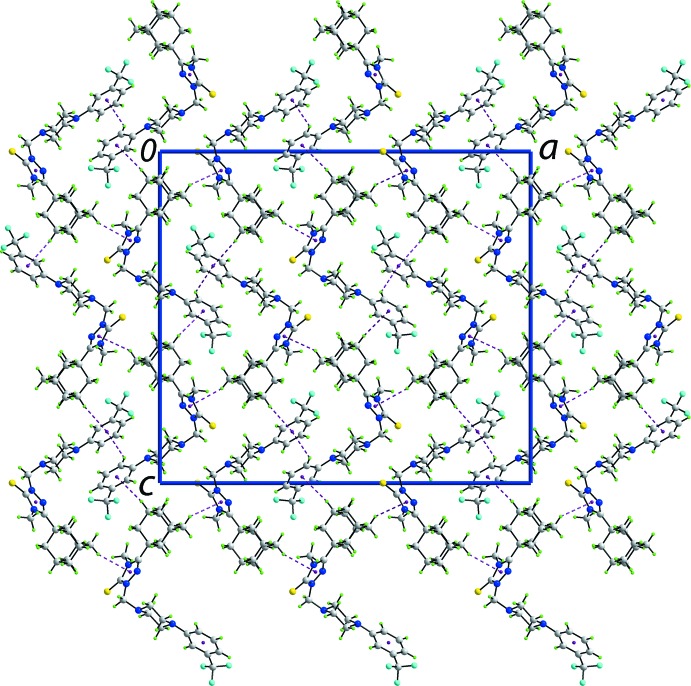
View of the unit-cell contents in projection down the b axis of (I). The C—H···π contacts are shown as purple dashed lines.

## supplementary crystallographic information

### Comment

In connection with the biological activities of adamantane derivatives (Al-Deeb *et al.*, 2006; Al-Omar *et al.*, 2010) and complementary structural studies (El-Emam *et al.*, 2012), the title compound was synthesized and characterized, including by X-ray crystallography.

Two independent molecules comprise the crystallographic asymmetric unit of (I), Fig. 1. The molecules are related across a pseudo centre of inversion. As shown in Fig. 2, the best fit between the molecules occurs when the inverted S2-containing molecule is superimposed upon the S1-containing molecule. Each piperazinyl ring has a chair conformation with the respective *N*-bound methylene and benzene ring substituents in equatorial positions. With respect to the triazole ring, the piperazinyl ring lies completely to one side with the N2—N3—C14—N4 torsion angle being -58.5 (5)°; for the S2-containing molecule, the equivalent N7—N8—C39—N9 torsion angle is 63.9 (5)°. The dihedral angles between the triazole and benzene rings are 78.20 (19) and 79.10 (19)° for the S1- and S2-containing molecules, respectively, so that overall, each molecule approximates the shape of the letter *L*.

The crystal packing of (I) is dominated by C—H···π interactions, Table 1, where the triazole and benzene rings of both independent molecules function as the π-systems; the benzene ring of the S1-containing molecule is bifurcated. These interactions result in a three-dimensional architecture, Fig. 3.

### Experimental

A mixture of 5-(adamantan-1-yl)-4-methyl-4*H*-1,2,4-triazole-3-thiol (499 mg, 2 mmol), 1-(3-trifluoromethylphenyl)piperazine (460 mg, 2 mmol) and 37% formaldehyde solution (1 ml), in ethanol (8 ml), was heated under reflux for 15 min. when a clear solution was obtained. Stirring was continued for 12 h at room temperature and the mixture was allowed to stand overnight. Cold water (5 ml) was slowly added and the mixture was stirred for 20 min. The precipitated crude product were filtered, washed with water, dried, and crystallized from aqueous ethanol to yield 551 mg (56%) of the title compound as colourless crystals. *M*.pt: 459–461 K. Single crystals suitable for X-ray analysis were obtained by slow evaporation of its CHCl_3_:EtOH solution (1:1, 5 ml) at room temperature. ^1^H NMR (CDCl_3_, 500.13 MHz): δ 1.76–1.84 (m, 6H, adamantane-H), 2.07 (s, 6H, adamantane-H), 2.13 (s, 3H, adamantane-H), 2.98 (s, 4H, piperazine-H), 3.24 (s, 4H, piperazine-H), 3.80 (s, 3H, CH_3_), 5.19 (s, 2H, CH_2_), 7.03–7.10 (m, 3H, Ar—H), 7.32–7.35 (m, 1H, Ar—H). ^13^C NMR (CDCl_3_, 125.76 MHz): δ 27.82, 35.10, 36.29, 39.02 (adamantane-C), 33.98 (CH_3_), 48.79, 50.21 (piperazine-C), 69.15 (CH_2_), 112.33, 115.89, 118.83, 123.21, 125.38, 129.54, 151.38 (Ar—C & CF_3_), 156.38 (triazole C-5), 169.58 (C═S).

### Refinement

The H-atoms were placed in calculated positions [and C—H = 0.93 to 0.98 Å, *U*_iso_(H) = 1.2–1.5*U*_eq_(C)] and were included in the refinement in the riding model approximation. The crystal is an inversion twin with the fractional contribution of the minor component being 0.27 (9).

### Crystal data

**Table:**

C_25_H_32_F_3_N_5_S	*F*(000) = 2080
*M**_r_* = 491.62	*D*_x_ = 1.332 Mg m^−^^3^
Orthorhombic, *P**n**a*2_1_	Mo *K*α radiation, λ = 0.71073 Å
Hall symbol: P 2c -2n	Cell parameters from 4723 reflections
*a* = 28.8100 (15) Å	θ = 2.9–27.5°
*b* = 6.6052 (4) Å	µ = 0.18 mm^−^^1^
*c* = 25.7717 (14) Å	*T* = 295 K
*V* = 4904.2 (5) Å^3^	Prism, colourless
*Z* = 8	0.40 × 0.30 × 0.20 mm

### Data collection

**Table:**

Agilent SuperNova Dual diffractometer with an Atlas detector	9351 independent reflections
Radiation source: SuperNova (Mo) X-ray Source	6701 reflections with *I* > 2σ(*I*)
Mirror monochromator	*R*_int_ = 0.038
Detector resolution: 10.4041 pixels mm^-1^	θ_max_ = 27.6°, θ_min_ = 2.9°
ω scan	*h* = −28→37
Absorption correction: multi-scan (*CrysAlis PRO*; Agilent, 2011)	*k* = −8→6
*T*_min_ = 0.878, *T*_max_ = 1.000	*l* = −27→33
19420 measured reflections	

### Refinement

**Table:**

Refinement on *F*^2^	Secondary atom site location: difference Fourier map
Least-squares matrix: full	Hydrogen site location: inferred from neighbouring sites
*R*[*F*^2^ > 2σ(*F*^2^)] = 0.058	H-atom parameters constrained
*wR*(*F*^2^) = 0.151	*w* = 1/[σ^2^(*F*_o_^2^) + (0.0676*P*)^2^ + 1.2433*P*] where *P* = (*F*_o_^2^ + 2*F*_c_^2^)/3
*S* = 1.03	(Δ/σ)_max_ = 0.001
9351 reflections	Δρ_max_ = 0.37 e Å^−^^3^
616 parameters	Δρ_min_ = −0.28 e Å^−^^3^
1 restraint	Absolute structure: Flack (1983), 3730 Friedel pairs
Primary atom site location: structure-invariant direct methods	Flack parameter: 0.27 (9)

### Special details

**Table:**

Geometry. All e.s.d.'s (except the e.s.d. in the dihedral angle between two l.s. planes) are estimated using the full covariance matrix. The cell e.s.d.'s are taken into account individually in the estimation of e.s.d.'s in distances, angles and torsion angles; correlations between e.s.d.'s in cell parameters are only used when they are defined by crystal symmetry. An approximate (isotropic) treatment of cell e.s.d.'s is used for estimating e.s.d.'s involving l.s. planes.
Refinement. Refinement of *F*^2^ against ALL reflections. The weighted *R*-factor *wR* and goodness of fit *S* are based on *F*^2^, conventional *R*-factors *R* are based on *F*, with *F* set to zero for negative *F*^2^. The threshold expression of *F*^2^ > σ(*F*^2^) is used only for calculating *R*-factors(gt) *etc*. and is not relevant to the choice of reflections for refinement. *R*-factors based on *F*^2^ are statistically about twice as large as those based on *F*, and *R*- factors based on ALL data will be even larger.

### Fractional atomic coordinates and isotropic or equivalent isotropic displacement parameters (Å^2^)

**Table:**

	*x*	*y*	*z*	*U*_iso_*/*U*_eq_	
S1	0.39860 (3)	0.38696 (19)	0.49983 (5)	0.0681 (3)	
S2	0.35800 (4)	0.5184 (2)	0.32727 (5)	0.0706 (3)	
F1	0.0788 (2)	0.6059 (8)	0.28978 (18)	0.183 (2)	
F2	0.13979 (13)	0.6066 (9)	0.2525 (2)	0.198 (3)	
F3	0.09032 (18)	0.8017 (6)	0.23096 (15)	0.1560 (18)	
F4	0.61728 (13)	0.4637 (5)	0.55280 (15)	0.1217 (13)	
F5	0.68603 (11)	0.3557 (6)	0.55014 (18)	0.1333 (15)	
F6	0.64004 (15)	0.2316 (6)	0.60229 (11)	0.1267 (13)	
N1	0.34008 (9)	0.4862 (4)	0.57964 (11)	0.0440 (6)	
N2	0.31554 (10)	0.7906 (5)	0.55884 (11)	0.0501 (7)	
N3	0.34609 (9)	0.7187 (5)	0.52206 (11)	0.0499 (7)	
N4	0.31612 (10)	0.8804 (5)	0.44287 (12)	0.0543 (8)	
N5	0.22650 (10)	0.9371 (4)	0.40013 (12)	0.0488 (7)	
N6	0.41461 (9)	0.4168 (4)	0.24638 (11)	0.0446 (6)	
N7	0.43862 (9)	0.1100 (5)	0.26586 (11)	0.0494 (7)	
N8	0.40902 (10)	0.1840 (5)	0.30367 (11)	0.0519 (7)	
N9	0.44187 (10)	0.0441 (5)	0.38380 (11)	0.0562 (8)	
N10	0.53329 (10)	0.0274 (4)	0.42481 (11)	0.0501 (7)	
C1	0.34866 (14)	0.2931 (6)	0.60558 (16)	0.0596 (10)	
H1A	0.3405	0.3043	0.6416	0.089*	
H1B	0.3809	0.2588	0.6026	0.089*	
H1C	0.3302	0.1894	0.5896	0.089*	
C2	0.31235 (11)	0.6474 (5)	0.59316 (13)	0.0431 (8)	
C3	0.36145 (11)	0.5316 (6)	0.53342 (13)	0.0459 (8)	
C4	0.28123 (10)	0.6645 (5)	0.64055 (13)	0.0398 (7)	
C5	0.30999 (12)	0.6808 (7)	0.69043 (14)	0.0539 (9)	
H5A	0.3308	0.7957	0.6881	0.065*	
H5B	0.3286	0.5595	0.6947	0.065*	
C6	0.27774 (15)	0.7067 (7)	0.73726 (15)	0.0646 (11)	
H6	0.2963	0.7153	0.7690	0.078*	
C7	0.24557 (16)	0.5274 (7)	0.74063 (17)	0.0689 (12)	
H7A	0.2256	0.5416	0.7707	0.083*	
H7B	0.2636	0.4045	0.7448	0.083*	
C8	0.21631 (14)	0.5120 (7)	0.69245 (17)	0.0632 (11)	
H8	0.1953	0.3957	0.6954	0.076*	
C9	0.24764 (13)	0.4852 (6)	0.64492 (16)	0.0565 (10)	
H9A	0.2288	0.4769	0.6138	0.068*	
H9B	0.2651	0.3602	0.6481	0.068*	
C10	0.25241 (14)	0.8580 (6)	0.63495 (18)	0.0643 (11)	
H10A	0.2337	0.8504	0.6037	0.077*	
H10B	0.2729	0.9739	0.6319	0.077*	
C11	0.22067 (15)	0.8846 (7)	0.68255 (19)	0.0694 (12)	
H11	0.2026	1.0093	0.6787	0.083*	
C12	0.24965 (16)	0.8978 (7)	0.7310 (2)	0.0770 (14)	
H12A	0.2296	0.9159	0.7610	0.092*	
H12B	0.2703	1.0136	0.7289	0.092*	
C13	0.18797 (13)	0.7053 (8)	0.68558 (19)	0.0738 (13)	
H13A	0.1697	0.6968	0.6541	0.089*	
H13B	0.1669	0.7219	0.7147	0.089*	
C14	0.35602 (12)	0.8363 (7)	0.47442 (15)	0.0600 (10)	
H14A	0.3705	0.9631	0.4843	0.072*	
H14B	0.3782	0.7614	0.4536	0.072*	
C15	0.28730 (13)	0.7071 (6)	0.43116 (15)	0.0571 (9)	
H15A	0.2700	0.6680	0.4619	0.068*	
H15B	0.3067	0.5938	0.4210	0.068*	
C16	0.25377 (14)	0.7573 (6)	0.38749 (15)	0.0595 (10)	
H16A	0.2710	0.7808	0.3557	0.071*	
H16B	0.2331	0.6436	0.3818	0.071*	
C17	0.25512 (14)	1.1091 (6)	0.41538 (18)	0.0636 (11)	
H17A	0.2353	1.2200	0.4264	0.076*	
H17B	0.2733	1.1546	0.3859	0.076*	
C18	0.28749 (15)	1.0502 (6)	0.45952 (17)	0.0609 (10)	
H18A	0.3071	1.1642	0.4687	0.073*	
H18B	0.2695	1.0123	0.4898	0.073*	
C19	0.18602 (12)	0.9753 (5)	0.37124 (13)	0.0464 (8)	
C20	0.15911 (13)	1.1469 (6)	0.38088 (14)	0.0595 (10)	
H20	0.1691	1.2408	0.4054	0.071*	
C21	0.11804 (15)	1.1798 (8)	0.35478 (17)	0.0749 (13)	
H21	0.1012	1.2969	0.3616	0.090*	
C22	0.10138 (14)	1.0445 (8)	0.31911 (16)	0.0702 (12)	
H22	0.0735	1.0676	0.3019	0.084*	
C23	0.12715 (12)	0.8732 (6)	0.30942 (13)	0.0507 (9)	
C24	0.16869 (11)	0.8385 (6)	0.33489 (14)	0.0480 (8)	
H24	0.1854	0.7214	0.3276	0.058*	
C25	0.10945 (14)	0.7250 (7)	0.27190 (18)	0.0629 (11)	
C26	0.40500 (15)	0.6074 (6)	0.21976 (18)	0.0656 (11)	
H26A	0.3728	0.6413	0.2238	0.098*	
H26B	0.4121	0.5935	0.1835	0.098*	
H26C	0.4238	0.7129	0.2345	0.098*	
C27	0.44172 (10)	0.2542 (5)	0.23169 (12)	0.0415 (7)	
C28	0.39408 (11)	0.3711 (6)	0.29321 (14)	0.0485 (8)	
C29	0.47076 (10)	0.2327 (5)	0.18353 (13)	0.0403 (7)	
C30	0.44003 (12)	0.2164 (6)	0.13534 (14)	0.0530 (9)	
H30A	0.4213	0.3376	0.1321	0.064*	
H30B	0.4193	0.1015	0.1389	0.064*	
C31	0.47015 (15)	0.1899 (7)	0.08613 (16)	0.0680 (11)	
H31	0.4499	0.1808	0.0556	0.082*	
C32	0.50187 (19)	0.3665 (7)	0.08024 (18)	0.0781 (13)	
H32A	0.4839	0.4901	0.0773	0.094*	
H32B	0.5202	0.3514	0.0489	0.094*	
C33	0.53413 (16)	0.3793 (7)	0.1277 (2)	0.0769 (14)	
H33	0.5553	0.4942	0.1235	0.092*	
C34	0.50505 (15)	0.4101 (6)	0.17700 (18)	0.0657 (11)	
H34A	0.4880	0.5364	0.1746	0.079*	
H34B	0.5253	0.4173	0.2070	0.079*	
C35	0.49929 (15)	0.0375 (6)	0.18721 (17)	0.0618 (10)	
H35A	0.4786	−0.0772	0.1911	0.074*	
H35B	0.5191	0.0432	0.2176	0.074*	
C36	0.52930 (14)	0.0085 (6)	0.13848 (18)	0.0639 (11)	
H36	0.5472	−0.1173	0.1415	0.077*	
C37	0.49783 (17)	−0.0028 (7)	0.09112 (17)	0.0698 (12)	
H37A	0.5165	−0.0226	0.0602	0.084*	
H37B	0.4769	−0.1171	0.0945	0.084*	
C38	0.56217 (13)	0.1856 (9)	0.1320 (2)	0.0845 (15)	
H38A	0.5829	0.1934	0.1615	0.101*	
H38B	0.5807	0.1672	0.1009	0.101*	
C39	0.40105 (13)	0.0682 (8)	0.35189 (15)	0.0659 (12)	
H39A	0.3773	0.1362	0.3721	0.079*	
H39B	0.3893	−0.0649	0.3428	0.079*	
C40	0.46762 (14)	0.2282 (6)	0.39268 (15)	0.0582 (10)	
H40A	0.4463	0.3359	0.4019	0.070*	
H40B	0.4834	0.2673	0.3610	0.070*	
C41	0.50309 (14)	0.2017 (6)	0.43576 (16)	0.0617 (10)	
H41A	0.5218	0.3233	0.4387	0.074*	
H41B	0.4872	0.1809	0.4685	0.074*	
C42	0.50745 (15)	−0.1556 (6)	0.41217 (17)	0.0639 (11)	
H42A	0.4910	−0.2022	0.4428	0.077*	
H42B	0.5289	−0.2610	0.4018	0.077*	
C43	0.47310 (14)	−0.1184 (6)	0.36896 (17)	0.0616 (10)	
H43A	0.4895	−0.0819	0.3374	0.074*	
H43B	0.4555	−0.2408	0.3623	0.074*	
C44	0.57378 (12)	0.0090 (5)	0.45413 (13)	0.0466 (8)	
C45	0.60424 (14)	−0.1533 (7)	0.44725 (15)	0.0598 (10)	
H45	0.5962	−0.2562	0.4243	0.072*	
C46	0.64559 (15)	−0.1659 (7)	0.47321 (16)	0.0683 (12)	
H46	0.6651	−0.2755	0.4670	0.082*	
C47	0.65905 (15)	−0.0202 (7)	0.50823 (17)	0.0679 (12)	
H47	0.6870	−0.0298	0.5261	0.082*	
C48	0.62912 (12)	0.1419 (6)	0.51580 (14)	0.0524 (9)	
C49	0.58751 (12)	0.1560 (6)	0.49019 (13)	0.0499 (9)	
H49	0.5680	0.2652	0.4969	0.060*	
C50	0.64274 (15)	0.2985 (7)	0.55468 (18)	0.0658 (11)	

### Atomic displacement parameters (Å^2^)

**Table:**

	*U*^11^	*U*^22^	*U*^33^	*U*^12^	*U*^13^	*U*^23^
S1	0.0566 (5)	0.0798 (8)	0.0680 (6)	0.0067 (6)	0.0152 (5)	−0.0190 (6)
S2	0.0606 (6)	0.0897 (8)	0.0616 (6)	0.0106 (6)	0.0147 (5)	−0.0182 (6)
F1	0.228 (5)	0.204 (5)	0.115 (3)	−0.144 (5)	0.026 (3)	−0.032 (3)
F2	0.109 (3)	0.234 (5)	0.250 (6)	0.041 (3)	−0.063 (3)	−0.187 (5)
F3	0.247 (5)	0.114 (3)	0.107 (3)	0.006 (3)	−0.109 (3)	−0.011 (2)
F4	0.136 (3)	0.092 (2)	0.138 (3)	0.040 (2)	−0.052 (3)	−0.048 (2)
F5	0.0796 (19)	0.145 (3)	0.175 (4)	−0.029 (2)	0.003 (2)	−0.068 (3)
F6	0.209 (4)	0.124 (3)	0.0473 (15)	−0.019 (3)	−0.009 (2)	−0.0154 (17)
N1	0.0397 (13)	0.0436 (14)	0.0487 (16)	0.0037 (13)	0.0001 (13)	−0.0005 (13)
N2	0.0447 (14)	0.0616 (18)	0.0440 (16)	0.0042 (15)	0.0044 (13)	0.0096 (15)
N3	0.0427 (13)	0.067 (2)	0.0398 (16)	0.0014 (15)	0.0041 (13)	0.0041 (15)
N4	0.0451 (15)	0.073 (2)	0.0451 (17)	0.0022 (16)	0.0006 (13)	0.0089 (16)
N5	0.0504 (15)	0.0474 (15)	0.0486 (16)	0.0036 (14)	−0.0003 (14)	−0.0005 (14)
N6	0.0432 (13)	0.0523 (16)	0.0383 (14)	0.0028 (14)	−0.0003 (12)	−0.0045 (13)
N7	0.0434 (14)	0.0614 (18)	0.0433 (15)	0.0054 (15)	0.0045 (13)	0.0061 (15)
N8	0.0443 (14)	0.073 (2)	0.0387 (16)	0.0004 (16)	0.0055 (13)	0.0053 (15)
N9	0.0479 (16)	0.078 (2)	0.0426 (16)	0.0069 (17)	0.0067 (14)	0.0122 (16)
N10	0.0546 (15)	0.0503 (16)	0.0454 (16)	0.0092 (15)	−0.0017 (14)	0.0004 (14)
C1	0.064 (2)	0.046 (2)	0.068 (3)	0.0124 (19)	0.013 (2)	0.0018 (19)
C2	0.0393 (15)	0.0481 (19)	0.0419 (17)	0.0028 (16)	−0.0043 (14)	0.0029 (16)
C3	0.0410 (17)	0.056 (2)	0.0410 (18)	−0.0010 (17)	−0.0038 (14)	−0.0044 (16)
C4	0.0366 (14)	0.0406 (17)	0.0421 (17)	0.0006 (15)	0.0018 (14)	0.0016 (14)
C5	0.0428 (17)	0.067 (2)	0.051 (2)	0.0029 (18)	−0.0044 (16)	−0.0075 (19)
C6	0.064 (2)	0.083 (3)	0.046 (2)	0.006 (2)	0.0008 (19)	−0.010 (2)
C7	0.087 (3)	0.071 (3)	0.049 (2)	0.014 (3)	0.022 (2)	0.011 (2)
C8	0.060 (2)	0.062 (2)	0.068 (3)	−0.016 (2)	0.025 (2)	−0.003 (2)
C9	0.0508 (19)	0.059 (2)	0.059 (2)	−0.0107 (19)	0.0053 (18)	−0.006 (2)
C10	0.058 (2)	0.060 (2)	0.075 (3)	0.022 (2)	0.017 (2)	0.019 (2)
C11	0.066 (2)	0.060 (2)	0.083 (3)	0.029 (2)	0.023 (2)	0.012 (2)
C12	0.074 (3)	0.067 (3)	0.091 (4)	−0.007 (3)	0.031 (3)	−0.029 (3)
C13	0.0418 (18)	0.109 (4)	0.071 (3)	0.012 (2)	0.012 (2)	0.006 (3)
C14	0.0447 (18)	0.087 (3)	0.048 (2)	0.001 (2)	0.0070 (17)	0.016 (2)
C15	0.058 (2)	0.069 (2)	0.0442 (19)	0.016 (2)	−0.0020 (18)	−0.0016 (19)
C16	0.057 (2)	0.067 (2)	0.055 (2)	0.012 (2)	−0.0048 (18)	−0.006 (2)
C17	0.059 (2)	0.056 (2)	0.077 (3)	−0.006 (2)	−0.007 (2)	0.011 (2)
C18	0.063 (2)	0.055 (2)	0.064 (2)	−0.002 (2)	−0.008 (2)	−0.001 (2)
C19	0.0498 (18)	0.054 (2)	0.0357 (17)	0.0056 (17)	0.0058 (15)	0.0063 (16)
C20	0.062 (2)	0.071 (2)	0.045 (2)	0.018 (2)	−0.0023 (18)	−0.0123 (19)
C21	0.075 (3)	0.087 (3)	0.063 (3)	0.036 (3)	−0.008 (2)	−0.010 (2)
C22	0.061 (2)	0.103 (3)	0.047 (2)	0.027 (3)	−0.007 (2)	0.001 (2)
C23	0.0509 (18)	0.064 (2)	0.0378 (17)	0.0008 (19)	0.0024 (15)	0.0036 (17)
C24	0.0486 (17)	0.0510 (19)	0.044 (2)	0.0026 (17)	0.0028 (16)	0.0032 (17)
C25	0.0488 (19)	0.075 (3)	0.065 (3)	0.003 (2)	−0.005 (2)	−0.002 (2)
C26	0.071 (2)	0.053 (2)	0.072 (3)	0.008 (2)	0.012 (2)	0.003 (2)
C27	0.0356 (15)	0.0478 (18)	0.0411 (17)	−0.0002 (16)	−0.0042 (14)	0.0005 (15)
C28	0.0376 (16)	0.067 (2)	0.0410 (18)	−0.0030 (18)	−0.0015 (14)	−0.0073 (18)
C29	0.0352 (14)	0.0461 (17)	0.0395 (17)	−0.0078 (15)	0.0025 (14)	−0.0009 (15)
C30	0.0486 (17)	0.064 (2)	0.046 (2)	−0.0010 (18)	−0.0008 (16)	−0.0075 (18)
C31	0.072 (2)	0.088 (3)	0.044 (2)	0.006 (3)	−0.002 (2)	−0.012 (2)
C32	0.102 (3)	0.075 (3)	0.057 (3)	0.013 (3)	0.035 (3)	0.010 (2)
C33	0.070 (3)	0.067 (3)	0.094 (4)	−0.032 (2)	0.038 (3)	−0.016 (3)
C34	0.062 (2)	0.065 (3)	0.070 (3)	−0.022 (2)	0.016 (2)	−0.019 (2)
C35	0.058 (2)	0.068 (3)	0.059 (2)	0.021 (2)	0.0180 (19)	0.014 (2)
C36	0.056 (2)	0.063 (2)	0.073 (3)	0.017 (2)	0.020 (2)	0.003 (2)
C37	0.080 (3)	0.065 (2)	0.065 (3)	−0.003 (2)	0.022 (2)	−0.017 (2)
C38	0.0426 (19)	0.125 (4)	0.086 (3)	−0.006 (3)	0.021 (2)	−0.014 (3)
C39	0.0468 (19)	0.102 (3)	0.048 (2)	0.005 (2)	0.0094 (17)	0.021 (2)
C40	0.061 (2)	0.068 (2)	0.046 (2)	0.021 (2)	−0.0017 (18)	−0.004 (2)
C41	0.060 (2)	0.077 (3)	0.048 (2)	0.026 (2)	−0.0053 (18)	−0.012 (2)
C42	0.065 (2)	0.056 (2)	0.070 (3)	−0.003 (2)	−0.003 (2)	0.012 (2)
C43	0.060 (2)	0.065 (2)	0.059 (2)	−0.002 (2)	−0.0061 (19)	0.001 (2)
C44	0.0502 (19)	0.055 (2)	0.0349 (16)	0.0071 (18)	0.0105 (14)	0.0031 (15)
C45	0.065 (2)	0.066 (2)	0.048 (2)	0.020 (2)	0.0000 (18)	−0.0140 (18)
C46	0.066 (2)	0.080 (3)	0.059 (2)	0.031 (2)	−0.003 (2)	−0.013 (2)
C47	0.060 (2)	0.087 (3)	0.057 (2)	0.022 (2)	−0.004 (2)	−0.012 (2)
C48	0.0524 (18)	0.062 (2)	0.0427 (19)	0.0056 (19)	−0.0013 (16)	−0.0028 (17)
C49	0.0551 (19)	0.052 (2)	0.0424 (19)	0.0124 (18)	0.0083 (16)	0.0015 (16)
C50	0.066 (3)	0.070 (3)	0.061 (3)	0.005 (2)	−0.005 (2)	−0.010 (2)

### Geometric parameters (Å, º)

**Table:**

S1—C3	1.676 (4)	C16—H16B	0.9700
S2—C28	1.673 (4)	C17—C18	1.522 (6)
F1—C25	1.270 (6)	C17—H17A	0.9700
F2—C25	1.275 (5)	C17—H17B	0.9700
F3—C25	1.294 (5)	C18—H18A	0.9700
F4—C50	1.316 (5)	C18—H18B	0.9700
F5—C50	1.309 (5)	C19—C20	1.395 (5)
F6—C50	1.307 (6)	C19—C24	1.394 (5)
N1—C3	1.374 (4)	C20—C21	1.378 (5)
N1—C2	1.376 (4)	C20—H20	0.9300
N1—C1	1.461 (5)	C21—C22	1.369 (6)
N2—C2	1.298 (4)	C21—H21	0.9300
N2—N3	1.378 (4)	C22—C23	1.376 (6)
N3—C3	1.345 (5)	C22—H22	0.9300
N3—C14	1.481 (5)	C23—C24	1.384 (5)
N4—C14	1.438 (5)	C23—C25	1.468 (6)
N4—C15	1.446 (5)	C24—H24	0.9300
N4—C18	1.457 (5)	C26—H26A	0.9600
N5—C19	1.407 (4)	C26—H26B	0.9600
N5—C17	1.457 (5)	C26—H26C	0.9600
N5—C16	1.461 (5)	C27—C29	1.503 (5)
N6—C28	1.378 (4)	C29—C30	1.529 (5)
N6—C27	1.381 (4)	C29—C35	1.532 (5)
N6—C26	1.460 (5)	C29—C34	1.542 (5)
N7—C27	1.300 (4)	C30—C31	1.547 (5)
N7—N8	1.384 (4)	C30—H30A	0.9700
N8—C28	1.336 (5)	C30—H30B	0.9700
N8—C39	1.477 (5)	C31—C32	1.489 (6)
N9—C40	1.443 (5)	C31—C37	1.508 (6)
N9—C39	1.444 (5)	C31—H31	0.9800
N9—C43	1.452 (5)	C32—C33	1.539 (7)
N10—C44	1.395 (4)	C32—H32A	0.9700
N10—C42	1.456 (5)	C32—H32B	0.9700
N10—C41	1.471 (5)	C33—C38	1.517 (7)
C1—H1A	0.9600	C33—C34	1.535 (6)
C1—H1B	0.9600	C33—H33	0.9800
C1—H1C	0.9600	C34—H34A	0.9700
C2—C4	1.519 (5)	C34—H34B	0.9700
C4—C10	1.531 (5)	C35—C36	1.537 (6)
C4—C5	1.533 (5)	C35—H35A	0.9700
C4—C9	1.534 (5)	C35—H35B	0.9700
C5—C6	1.533 (5)	C36—C38	1.514 (6)
C5—H5A	0.9700	C36—C37	1.522 (6)
C5—H5B	0.9700	C36—H36	0.9800
C6—C7	1.507 (6)	C37—H37A	0.9700
C6—C12	1.508 (6)	C37—H37B	0.9700
C6—H6	0.9800	C38—H38A	0.9700
C7—C8	1.504 (6)	C38—H38B	0.9700
C7—H7A	0.9700	C39—H39A	0.9700
C7—H7B	0.9700	C39—H39B	0.9700
C8—C13	1.526 (6)	C40—C41	1.519 (5)
C8—C9	1.532 (5)	C40—H40A	0.9700
C8—H8	0.9800	C40—H40B	0.9700
C9—H9A	0.9700	C41—H41A	0.9700
C9—H9B	0.9700	C41—H41B	0.9700
C10—C11	1.540 (6)	C42—C43	1.510 (6)
C10—H10A	0.9700	C42—H42A	0.9700
C10—H10B	0.9700	C42—H42B	0.9700
C11—C12	1.505 (7)	C43—H43A	0.9700
C11—C13	1.516 (6)	C43—H43B	0.9700
C11—H11	0.9800	C44—C45	1.397 (5)
C12—H12A	0.9700	C44—C49	1.400 (5)
C12—H12B	0.9700	C45—C46	1.369 (6)
C13—H13A	0.9700	C45—H45	0.9300
C13—H13B	0.9700	C46—C47	1.375 (6)
C14—H14A	0.9700	C46—H46	0.9300
C14—H14B	0.9700	C47—C48	1.388 (6)
C15—C16	1.520 (5)	C47—H47	0.9300
C15—H15A	0.9700	C48—C49	1.372 (5)
C15—H15B	0.9700	C48—C50	1.493 (6)
C16—H16A	0.9700	C49—H49	0.9300
			
C3—N1—C2	108.1 (3)	C23—C22—H22	121.0
C3—N1—C1	120.8 (3)	C22—C23—C24	121.1 (4)
C2—N1—C1	131.1 (3)	C22—C23—C25	118.7 (3)
C2—N2—N3	105.2 (3)	C24—C23—C25	120.1 (4)
C3—N3—N2	112.1 (3)	C23—C24—C19	121.4 (3)
C3—N3—C14	126.8 (3)	C23—C24—H24	119.3
N2—N3—C14	120.9 (3)	C19—C24—H24	119.3
C14—N4—C15	114.6 (3)	F1—C25—F2	103.9 (5)
C14—N4—C18	116.2 (3)	F1—C25—F3	104.0 (4)
C15—N4—C18	110.2 (3)	F2—C25—F3	102.2 (5)
C19—N5—C17	118.2 (3)	F1—C25—C23	114.6 (4)
C19—N5—C16	118.3 (3)	F2—C25—C23	115.4 (3)
C17—N5—C16	112.9 (3)	F3—C25—C23	115.1 (4)
C28—N6—C27	108.2 (3)	N6—C26—H26A	109.5
C28—N6—C26	121.3 (3)	N6—C26—H26B	109.5
C27—N6—C26	130.5 (3)	H26A—C26—H26B	109.5
C27—N7—N8	105.1 (3)	N6—C26—H26C	109.5
C28—N8—N7	112.5 (3)	H26A—C26—H26C	109.5
C28—N8—C39	126.8 (3)	H26B—C26—H26C	109.5
N7—N8—C39	120.4 (3)	N7—C27—N6	110.2 (3)
C40—N9—C39	114.6 (3)	N7—C27—C29	121.9 (3)
C40—N9—C43	110.3 (3)	N6—C27—C29	127.9 (3)
C39—N9—C43	115.9 (3)	N8—C28—N6	104.0 (3)
C44—N10—C42	118.5 (3)	N8—C28—S2	129.2 (3)
C44—N10—C41	117.3 (3)	N6—C28—S2	126.8 (3)
C42—N10—C41	113.0 (3)	C27—C29—C30	110.8 (2)
N1—C1—H1A	109.5	C27—C29—C35	109.1 (3)
N1—C1—H1B	109.5	C30—C29—C35	107.5 (3)
H1A—C1—H1B	109.5	C27—C29—C34	112.0 (3)
N1—C1—H1C	109.5	C30—C29—C34	109.6 (3)
H1A—C1—H1C	109.5	C35—C29—C34	107.6 (3)
H1B—C1—H1C	109.5	C29—C30—C31	110.4 (3)
N2—C2—N1	110.5 (3)	C29—C30—H30A	109.6
N2—C2—C4	122.4 (3)	C31—C30—H30A	109.6
N1—C2—C4	127.2 (3)	C29—C30—H30B	109.6
N3—C3—N1	104.0 (3)	C31—C30—H30B	109.6
N3—C3—S1	128.4 (3)	H30A—C30—H30B	108.1
N1—C3—S1	127.6 (3)	C32—C31—C37	110.2 (4)
C2—C4—C10	107.9 (3)	C32—C31—C30	109.8 (3)
C2—C4—C5	111.1 (2)	C37—C31—C30	108.8 (4)
C10—C4—C5	108.3 (3)	C32—C31—H31	109.3
C2—C4—C9	112.0 (3)	C37—C31—H31	109.3
C10—C4—C9	108.0 (3)	C30—C31—H31	109.3
C5—C4—C9	109.5 (3)	C31—C32—C33	109.4 (4)
C4—C5—C6	109.9 (3)	C31—C32—H32A	109.8
C4—C5—H5A	109.7	C33—C32—H32A	109.8
C6—C5—H5A	109.7	C31—C32—H32B	109.8
C4—C5—H5B	109.7	C33—C32—H32B	109.8
C6—C5—H5B	109.7	H32A—C32—H32B	108.2
H5A—C5—H5B	108.2	C38—C33—C32	109.4 (4)
C7—C6—C12	109.5 (3)	C38—C33—C34	110.0 (4)
C7—C6—C5	109.3 (3)	C32—C33—C34	109.6 (3)
C12—C6—C5	109.6 (4)	C38—C33—H33	109.3
C7—C6—H6	109.5	C32—C33—H33	109.3
C12—C6—H6	109.5	C34—C33—H33	109.3
C5—C6—H6	109.5	C33—C34—C29	109.9 (3)
C6—C7—C8	110.5 (3)	C33—C34—H34A	109.7
C6—C7—H7A	109.5	C29—C34—H34A	109.7
C8—C7—H7A	109.5	C33—C34—H34B	109.7
C6—C7—H7B	109.5	C29—C34—H34B	109.7
C8—C7—H7B	109.5	H34A—C34—H34B	108.2
H7A—C7—H7B	108.1	C29—C35—C36	110.9 (3)
C7—C8—C13	109.8 (4)	C29—C35—H35A	109.5
C7—C8—C9	109.7 (3)	C36—C35—H35A	109.5
C13—C8—C9	108.6 (4)	C29—C35—H35B	109.5
C7—C8—H8	109.6	C36—C35—H35B	109.5
C13—C8—H8	109.6	H35A—C35—H35B	108.1
C9—C8—H8	109.6	C38—C36—C37	108.7 (4)
C8—C9—C4	110.0 (3)	C38—C36—C35	110.3 (4)
C8—C9—H9A	109.7	C37—C36—C35	109.0 (3)
C4—C9—H9A	109.7	C38—C36—H36	109.6
C8—C9—H9B	109.7	C37—C36—H36	109.6
C4—C9—H9B	109.7	C35—C36—H36	109.6
H9A—C9—H9B	108.2	C31—C37—C36	110.0 (3)
C4—C10—C11	110.0 (3)	C31—C37—H37A	109.7
C4—C10—H10A	109.7	C36—C37—H37A	109.7
C11—C10—H10A	109.7	C31—C37—H37B	109.7
C4—C10—H10B	109.7	C36—C37—H37B	109.7
C11—C10—H10B	109.7	H37A—C37—H37B	108.2
H10A—C10—H10B	108.2	C33—C38—C36	109.1 (3)
C12—C11—C13	110.3 (4)	C33—C38—H38A	109.9
C12—C11—C10	109.8 (3)	C36—C38—H38A	109.9
C13—C11—C10	108.7 (4)	C33—C38—H38B	109.9
C12—C11—H11	109.3	C36—C38—H38B	109.9
C13—C11—H11	109.3	H38A—C38—H38B	108.3
C10—C11—H11	109.3	N9—C39—N8	114.2 (3)
C11—C12—C6	109.7 (3)	N9—C39—H39A	108.7
C11—C12—H12A	109.7	N8—C39—H39A	108.7
C6—C12—H12A	109.7	N9—C39—H39B	108.7
C11—C12—H12B	109.7	N8—C39—H39B	108.7
C6—C12—H12B	109.7	H39A—C39—H39B	107.6
H12A—C12—H12B	108.2	N9—C40—C41	111.4 (3)
C11—C13—C8	109.1 (3)	N9—C40—H40A	109.4
C11—C13—H13A	109.9	C41—C40—H40A	109.4
C8—C13—H13A	109.9	N9—C40—H40B	109.4
C11—C13—H13B	109.9	C41—C40—H40B	109.4
C8—C13—H13B	109.9	H40A—C40—H40B	108.0
H13A—C13—H13B	108.3	N10—C41—C40	110.4 (3)
N4—C14—N3	114.9 (3)	N10—C41—H41A	109.6
N4—C14—H14A	108.6	C40—C41—H41A	109.6
N3—C14—H14A	108.6	N10—C41—H41B	109.6
N4—C14—H14B	108.6	C40—C41—H41B	109.6
N3—C14—H14B	108.6	H41A—C41—H41B	108.1
H14A—C14—H14B	107.5	N10—C42—C43	111.4 (3)
N4—C15—C16	110.3 (3)	N10—C42—H42A	109.3
N4—C15—H15A	109.6	C43—C42—H42A	109.3
C16—C15—H15A	109.6	N10—C42—H42B	109.3
N4—C15—H15B	109.6	C43—C42—H42B	109.3
C16—C15—H15B	109.6	H42A—C42—H42B	108.0
H15A—C15—H15B	108.1	N9—C43—C42	109.4 (3)
N5—C16—C15	110.8 (3)	N9—C43—H43A	109.8
N5—C16—H16A	109.5	C42—C43—H43A	109.8
C15—C16—H16A	109.5	N9—C43—H43B	109.8
N5—C16—H16B	109.5	C42—C43—H43B	109.8
C15—C16—H16B	109.5	H43A—C43—H43B	108.2
H16A—C16—H16B	108.1	N10—C44—C45	121.5 (3)
N5—C17—C18	110.4 (3)	N10—C44—C49	122.4 (3)
N5—C17—H17A	109.6	C45—C44—C49	116.0 (3)
C18—C17—H17A	109.6	C46—C45—C44	122.1 (4)
N5—C17—H17B	109.6	C46—C45—H45	119.0
C18—C17—H17B	109.6	C44—C45—H45	119.0
H17A—C17—H17B	108.1	C45—C46—C47	121.6 (4)
N4—C18—C17	108.9 (3)	C45—C46—H46	119.2
N4—C18—H18A	109.9	C47—C46—H46	119.2
C17—C18—H18A	109.9	C46—C47—C48	117.2 (4)
N4—C18—H18B	109.9	C46—C47—H47	121.4
C17—C18—H18B	109.9	C48—C47—H47	121.4
H18A—C18—H18B	108.3	C49—C48—C47	121.8 (4)
C20—C19—C24	116.6 (3)	C49—C48—C50	120.4 (4)
C20—C19—N5	120.8 (3)	C47—C48—C50	117.7 (3)
C24—C19—N5	122.4 (3)	C48—C49—C44	121.3 (3)
C21—C20—C19	121.2 (4)	C48—C49—H49	119.4
C21—C20—H20	119.4	C44—C49—H49	119.4
C19—C20—H20	119.4	F6—C50—F5	103.8 (4)
C22—C21—C20	121.7 (4)	F6—C50—F4	106.4 (4)
C22—C21—H21	119.1	F5—C50—F4	106.8 (4)
C20—C21—H21	119.1	F6—C50—C48	112.4 (4)
C21—C22—C23	118.0 (4)	F5—C50—C48	113.0 (4)
C21—C22—H22	121.0	F4—C50—C48	113.8 (4)
			
C2—N2—N3—C3	0.4 (4)	C22—C23—C25—F3	40.1 (6)
C2—N2—N3—C14	175.8 (3)	C24—C23—C25—F3	−140.8 (4)
C27—N7—N8—C28	−0.1 (4)	N8—N7—C27—N6	−0.6 (4)
C27—N7—N8—C39	−173.7 (3)	N8—N7—C27—C29	−179.5 (3)
N3—N2—C2—N1	0.3 (4)	C28—N6—C27—N7	1.1 (4)
N3—N2—C2—C4	−179.2 (3)	C26—N6—C27—N7	−176.5 (4)
C3—N1—C2—N2	−0.8 (4)	C28—N6—C27—C29	179.9 (3)
C1—N1—C2—N2	179.2 (4)	C26—N6—C27—C29	2.3 (6)
C3—N1—C2—C4	178.6 (3)	N7—N8—C28—N6	0.8 (4)
C1—N1—C2—C4	−1.3 (6)	C39—N8—C28—N6	173.8 (3)
N2—N3—C3—N1	−0.9 (4)	N7—N8—C28—S2	179.6 (3)
C14—N3—C3—N1	−175.9 (3)	C39—N8—C28—S2	−7.4 (5)
N2—N3—C3—S1	179.9 (3)	C27—N6—C28—N8	−1.1 (3)
C14—N3—C3—S1	4.9 (5)	C26—N6—C28—N8	176.8 (3)
C2—N1—C3—N3	1.0 (3)	C27—N6—C28—S2	−179.9 (3)
C1—N1—C3—N3	−179.1 (3)	C26—N6—C28—S2	−2.0 (5)
C2—N1—C3—S1	−179.7 (3)	N7—C27—C29—C30	110.0 (4)
C1—N1—C3—S1	0.2 (5)	N6—C27—C29—C30	−68.7 (4)
N2—C2—C4—C10	7.0 (4)	N7—C27—C29—C35	−8.2 (4)
N1—C2—C4—C10	−172.3 (3)	N6—C27—C29—C35	173.1 (3)
N2—C2—C4—C5	−111.5 (4)	N7—C27—C29—C34	−127.2 (4)
N1—C2—C4—C5	69.1 (4)	N6—C27—C29—C34	54.1 (4)
N2—C2—C4—C9	125.7 (4)	C27—C29—C30—C31	−178.8 (3)
N1—C2—C4—C9	−53.6 (4)	C35—C29—C30—C31	−59.7 (4)
C2—C4—C5—C6	177.7 (3)	C34—C29—C30—C31	57.0 (4)
C10—C4—C5—C6	59.4 (4)	C29—C30—C31—C32	−59.6 (5)
C9—C4—C5—C6	−58.2 (4)	C29—C30—C31—C37	61.1 (5)
C4—C5—C6—C7	59.4 (4)	C37—C31—C32—C33	−59.0 (4)
C4—C5—C6—C12	−60.6 (4)	C30—C31—C32—C33	60.8 (5)
C12—C6—C7—C8	59.4 (4)	C31—C32—C33—C38	59.5 (4)
C5—C6—C7—C8	−60.6 (4)	C31—C32—C33—C34	−61.2 (5)
C6—C7—C8—C13	−59.0 (4)	C38—C33—C34—C29	−61.4 (5)
C6—C7—C8—C9	60.3 (4)	C32—C33—C34—C29	59.0 (5)
C7—C8—C9—C4	−58.6 (4)	C27—C29—C34—C33	179.3 (3)
C13—C8—C9—C4	61.4 (4)	C30—C29—C34—C33	−57.2 (4)
C2—C4—C9—C8	−178.6 (3)	C35—C29—C34—C33	59.5 (4)
C10—C4—C9—C8	−60.0 (4)	C27—C29—C35—C36	179.6 (3)
C5—C4—C9—C8	57.7 (4)	C30—C29—C35—C36	59.3 (4)
C2—C4—C10—C11	−179.1 (3)	C34—C29—C35—C36	−58.7 (4)
C5—C4—C10—C11	−58.8 (4)	C29—C35—C36—C38	59.5 (5)
C9—C4—C10—C11	59.7 (4)	C29—C35—C36—C37	−59.8 (5)
C4—C10—C11—C12	59.7 (5)	C32—C31—C37—C36	60.1 (4)
C4—C10—C11—C13	−61.1 (4)	C30—C31—C37—C36	−60.4 (4)
C13—C11—C12—C6	59.9 (4)	C38—C36—C37—C31	−60.3 (4)
C10—C11—C12—C6	−59.9 (5)	C35—C36—C37—C31	59.9 (4)
C7—C6—C12—C11	−59.4 (5)	C32—C33—C38—C36	−60.4 (5)
C5—C6—C12—C11	60.5 (4)	C34—C33—C38—C36	60.1 (5)
C12—C11—C13—C8	−58.9 (5)	C37—C36—C38—C33	60.7 (5)
C10—C11—C13—C8	61.6 (5)	C35—C36—C38—C33	−58.8 (5)
C7—C8—C13—C11	58.1 (5)	C40—N9—C39—N8	48.1 (5)
C9—C8—C13—C11	−61.9 (5)	C43—N9—C39—N8	−82.0 (5)
C15—N4—C14—N3	−50.2 (5)	C28—N8—C39—N9	−108.7 (4)
C18—N4—C14—N3	80.4 (5)	N7—N8—C39—N9	63.9 (5)
C3—N3—C14—N4	116.2 (4)	C39—N9—C40—C41	167.1 (3)
N2—N3—C14—N4	−58.5 (5)	C43—N9—C40—C41	−60.1 (4)
C14—N4—C15—C16	−165.8 (3)	C44—N10—C41—C40	166.1 (3)
C18—N4—C15—C16	60.7 (4)	C42—N10—C41—C40	−50.6 (4)
C19—N5—C16—C15	−163.7 (3)	N9—C40—C41—N10	54.0 (4)
C17—N5—C16—C15	52.3 (4)	C44—N10—C42—C43	−164.2 (3)
N4—C15—C16—N5	−54.9 (4)	C41—N10—C42—C43	53.0 (4)
C19—N5—C17—C18	161.9 (3)	C40—N9—C43—C42	60.8 (4)
C16—N5—C17—C18	−54.0 (4)	C39—N9—C43—C42	−167.0 (3)
C14—N4—C18—C17	165.5 (3)	N10—C42—C43—N9	−57.1 (4)
C15—N4—C18—C17	−61.9 (4)	C42—N10—C44—C45	37.6 (5)
N5—C17—C18—N4	57.8 (4)	C41—N10—C44—C45	178.8 (4)
C17—N5—C19—C20	−36.5 (5)	C42—N10—C44—C49	−145.6 (4)
C16—N5—C19—C20	−178.7 (3)	C41—N10—C44—C49	−4.4 (5)
C17—N5—C19—C24	148.7 (4)	N10—C44—C45—C46	175.4 (4)
C16—N5—C19—C24	6.6 (5)	C49—C44—C45—C46	−1.6 (6)
C24—C19—C20—C21	−1.3 (6)	C44—C45—C46—C47	1.1 (7)
N5—C19—C20—C21	−176.3 (4)	C45—C46—C47—C48	−0.7 (7)
C19—C20—C21—C22	1.3 (7)	C46—C47—C48—C49	0.9 (6)
C20—C21—C22—C23	−0.6 (7)	C46—C47—C48—C50	178.5 (4)
C21—C22—C23—C24	0.0 (6)	C47—C48—C49—C44	−1.5 (6)
C21—C22—C23—C25	179.1 (4)	C50—C48—C49—C44	−179.1 (4)
C22—C23—C24—C19	−0.1 (5)	N10—C44—C49—C48	−175.2 (3)
C25—C23—C24—C19	−179.1 (4)	C45—C44—C49—C48	1.8 (5)
C20—C19—C24—C23	0.7 (5)	C49—C48—C50—F6	106.8 (5)
N5—C19—C24—C23	175.7 (3)	C47—C48—C50—F6	−70.8 (5)
C22—C23—C25—F1	−80.4 (6)	C49—C48—C50—F5	−136.2 (4)
C24—C23—C25—F1	98.6 (5)	C47—C48—C50—F5	46.2 (6)
C22—C23—C25—F2	158.9 (5)	C49—C48—C50—F4	−14.2 (6)
C24—C23—C25—F2	−22.0 (7)	C47—C48—C50—F4	168.2 (4)

### Hydrogen-bond geometry (Å, º)

Cg1–Cg4 are the centroids of the N1–N3,C2,C3, C19–C24, N6–N8,C27,C28 and C44–C49 rings, respectively.

**Table:**

*D*—H···*A*	*D*—H	H···*A*	*D*···*A*	*D*—H···*A*
C6—H6···*Cg*2^i^	0.98	2.95	3.817 (4)	148
C13—H13*B*···*Cg*3^ii^	0.97	2.86	3.782 (4)	158
C31—H31···*Cg*4^iii^	0.98	2.94	3.873 (5)	159
C38—H38*B*···*Cg*1^iv^	0.97	2.97	3.723 (5)	135
C45—H45···*Cg*2^v^	0.93	2.97	3.708 (5)	137

Symmetry codes: (i) *x*+1/2, −*y*−1/2, *z*; (ii) *x*+1/2, −*y*+1/2, *z*; (iii) −*x*+1, −*y*, *z*−1/2; (iv) −*x*+1, −*y*+1, *z*−1/2; (v) −*x*+1/2, *y*+1/2, *z*+1/2.
